# Mechanical signatures in cancer metastasis

**DOI:** 10.1038/s44341-024-00007-x

**Published:** 2025-02-04

**Authors:** Ayushi Agrawal, Yousef Javanmardi, Sara A. Watson, Bianca Serwinski, Boris Djordjevic, Wenbin Li, Amir R. Aref, Russell W. Jenkins, Emad Moeendarbary

**Affiliations:** 1 Department of Mechanical Engineering, University College London, London, UK; 2 Division of Biosciences, University College London, London, UK; 3 Northeastern University London, London, UK; 4 Department of Neuro-Oncology, Cancer Center, Beijing Tiantan Hospital, Capital Medical University, Beijing, China; 5 Department of Surgery, Massachusetts General Hospital, Harvard Medical School, Boston, MA USA; 6 Massachusetts General Hospital Cancer Center, Department of Medicine, Massachusetts General Hospital, Harvard Medical School, Boston, MA USA; 7 Broad Institute of MIT and Harvard, Cambridge, MA USA; 8 Department of Biological Engineering, Massachusetts Institute of Technology, Cambridge, MA USA

**Keywords:** Biological physics, Diseases

## Abstract

The cancer metastatic cascade includes a series of mechanical barrier-crossing events, involving the physical movement of cancer cells from their primary location to a distant organ. This review describes the physical changes that influence tumour proliferation, progression, and metastasis. We identify potential mechanical signatures at every step of the metastatic cascade and discuss some latest mechanobiology-based therapeutic interventions to highlight the importance of interdisciplinary approaches in cancer diagnosis and treatment.

## Introduction

Cancer metastasis is a major cause of cancer-related deaths characterised by the physical movement of malignant cells from a primary tumour site to a distant tissue. The outgrowth of cancer cells has been widely studied from the perspectives of its genetic^1^, proteomic^2^, and epigenetic factors^3^. However, previous studies have largely ignored the mechanical cues that are intricately interwoven with these factors, and whose importance has become increasingly apparent over the past two decades. Several mechanical factors influence cancer metastasis, such as intracellular tension within the tumour, the biophysical properties of the tumour microenvironment (TME), contractility-induced stress by tumour stromal cells, the elastic resistance of the extracellular matrix (ECM), interstitial fluid pressure (IFP), the physical interactions of cancer cells with vasculature by crossing the endothelial barrier, withstanding forces of blood flow, and the physical properties of the secondary site.

Cells sense mechanical cues produced by the direct application of forces (e.g. tensile, compressive, pressure, and shear forces) or indirectly through changes in the structural and mechanical properties of the extracellular environment (such as the stiffness or microstructural architecture of the ECM)^4,5^. These mechanical stimuli trigger a dynamic rearrangement of the intracellular cytoskeleton, which can enhance either its rigidity or its deformability. Through this rearrangement, the cytoskeleton transitions from a rigid or ordered state to an irregular or compliant framework capable of translocating from a cytoplasmic to a perinuclear location in the presence of force. Thus, cells respond to mechanical cues by changing their internal microstructure as well as by inducing biophysical changes to the ECM, including its geometry, mechanics, and topology^6^. Further interactions with the ECM can subsequently change the cellular mechanical behaviour through an interconnected hierarchy of mechanochemical systems including adhesion receptors (e.g. integrin), intracellular focal adhesions (e.g. FAK), cytoskeletal networks (e.g. actin) and molecular motors (e.g. myosin)^7^. These systems induce mechanotransduction pathways in cancer through ERK activation, cytoskeletal remodelling, Rho-GTPase-dependent contractility, and integrin clustering^6^. This establishes a dynamic mechanical interplay between the cell and the ECM that ultimately influences tissue morphology. Abnormalities in cell-cell and cell-ECM adhesion and in cytoskeleton remodelling lead the cancer cell to develop an invasive morphology capable of invading through the ECM, thereby beginning the metastatic cascade^8^.

**Fig. 1:**
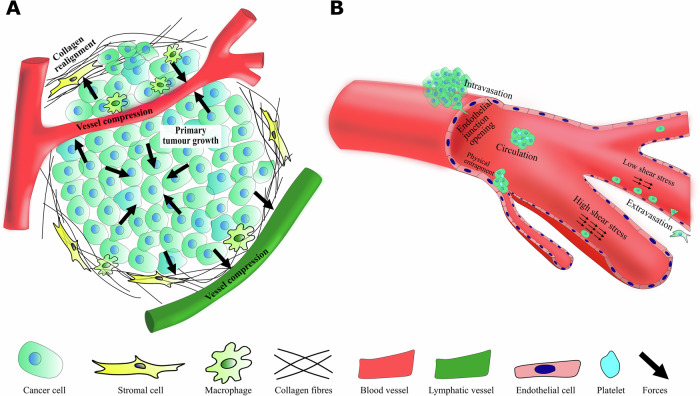
The Metastatic cascade. Metastatic cascade featuring various stages of cancer dissemination along with a few diverse sources of stress generation in the tumour and its microenvironment. **a** primary tumour growth and the onset of invasion. The solid stress in the tumour interior is mostly compressive, and mostly tensile at its periphery near the normal tissue. The solid stress at the tumour periphery thus compresses the surrounding tissue including the ECM, stromal cells, blood and lymphatic vessels. The solid stress is mostly compressive in the tumour interior (inward-pointing arrows) and mostly tensile at its periphery (outward-pointing arrows) near the normal tissue. The solid stress at the tumour periphery thus compresses the surrounding tissue, which includes the ECM, stromal cells, and blood and lymphatic vessels. **b** intravasation, circulation, and extravasation of cancer cells. The level of fluid shear stress controls the efficiency of adhesion of the cancer cells to the endothelial cells. High fluid shear prevents cell attachment while low fluid shear favours cell attachment. CTC clusters become physically entrapped to interact and adhere to the endothelium. The stresses within the tumour microenvironment are depicted by black arrows.

The metastatic cascade (Fig. 1) is a sequence of steps that leads to cancer metastasis, beginning with the malignant transformation of a primary tumour into an invasive phenotype that can invade the local tissue. This subsequently leads to malignant cells entering blood or lymphatic vessels via transendothelial migration (cancer-cell intravasation^9^). Once cancer cells enter the vasculature, they must withstand the forces of blood circulation and evade immune surveillance. Circulating tumour cells (CTCs) then adhere or become physically trapped in a remote microvascular network and migrate from the vessel lumen to surrounding tissues by transendothelial migration (cancer-cell extravasation). Finally, cancer cells localise to a distant tissue and proliferate to form secondary tumours (colonisation). The successful colonisation of a secondary site highlights the cancer cell’s adaptations to several physical stresses, such as a stiffened ECM and a basement membrane (BM), tight junctions, and fluid shear stress, all of which alter the cellular mechanical phenotype. Hence, mechanical sensing, mechanotransduction processes, and mechanical reciprocity play a central role at all stages of the metastatic cascade, including cancer initiation, progression, and propagation. Thus, to develop new and efficient modalities for cancer diagnosis, prevention, and treatment, a fundamental understanding of metastasis must be obtained, not only from the genetic, molecular, and biochemical bases but also from the main mechanical characteristics.

**Fig. 2:**
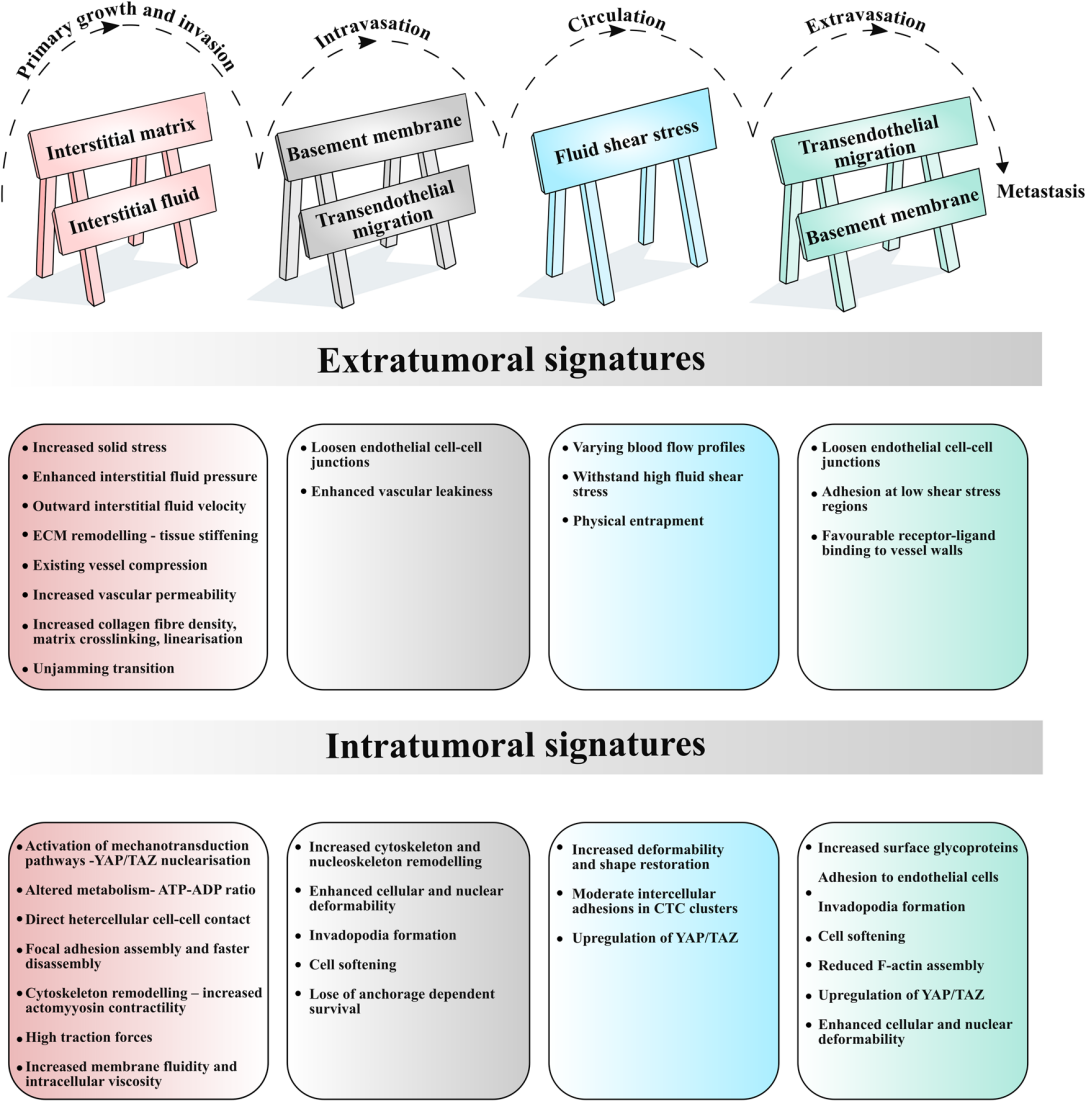
Mechanical signatures in the metastatic cascade. Summary of the key mechanical signatures at the intra- (within the cancer cell) and extratumoural (everything surrounding the cancer cells) levels. The barricades at the top of the figure symbolise the physical barriers to cancer progression through the metastatic cascade. Interstitial fluid and blood flow could act as mechanical barriers hindering the displacement of cancer cells. Cancer cells must endure significant fluid shear stress while circulating through the bloodstream.

This review discusses and highlights the importance of the mechanical cues that cancer cells encounter and their response at every step of the metastatic cascade. The key mechanical signatures throughout the stages of metastasis have been identified and are summarised in Fig. 2. We further investigate the existing tools and techniques used by researchers for testing cell and tissue mechanical properties. We also describe the concerns associated with measurement accuracy and the appropriateness of the research methodology used to ascertain the mechanical data. Then, current and future clinical implications with a focus on biomechanical alterations are also reviewed.

## Mechanical modulators of the tumour microenvironment

During their transformation, cancer cells dwell in a complex microenvironment composed of other cells, blood^10^ and lymphatic vessels, acellular components (e.g. the ECM the basement membrane), and soluble molecules (e.g. growth factors, cytokines, and chemokines) that diffuse in the interstitial fluids^11^. Although the initiation of cancer is unambiguously driven by genetic alterations to oncogenes and tumour suppressors, the TME plays a major role in influencing the lifespan of oncogenic cells^12^. In addition, the microenvironment also influences primary tumour growth, migration from the primary site, metastatic foci, and drug resistance^11^. Studies have established the key importance of numerous cellular and acellular factors in the TME, ranging from immune cells^13^, such as T cells, natural killer (NK) cells, macrophages, and stromal cells including mesenchymal cells, fibroblasts^14^, normal epithelial cells, and adipocytes; as well as acellular environmental factors, such as the ECM stiffness^15^, hypoxia^16^, and interstitial pressure^17^.

## Selective pressure in the tumour microenvironment

During the primary stage of tumour growth, when cancer cells undergo uncontrolled proliferation, the expanding tumour mass generates intra- and extratumoural mechanical forces. These forces cause irregular solid and fluid stresses that promote tumour progression and hamper responses to various treatments. Solid stress accumulates within tumour owing to the rapid proliferation of cancer cells that strains the TME as the growing mass pushes against itself, causing a high cell packing density and deforming the surrounding normal tissue^18^. In turn, the native surrounding tissue resists deformation due to tumour expansion by applying additional compressive stress. The total solid stress in the tumour interior is mostly compressive (i.e. tending to reduce tumour size), but mostly tensile at its periphery near the normal tissue (i.e. tending to increase tumour size)^15^. This produces heterogeneous proliferation rates across the tumour, with compressive stress at the centre usually resulting in cell-cycle arrest^19^. Volumetric compression has been shown to drive transcriptomic and phenotypic adaptations in malignant melanoma cells; increased pigmentation and resistance to cisplatin treatment have also been observed^20^. These behaviours were also apparent in compressed liver cancer cells that acquired chemotherapeutic resistance through impaired calcium signalling, and that were subsequently rescued by stimulated calcium mobilisation^21^. Similarly, solid stresses at the tumour periphery compresses the ECM, stromal cells, lymphatic, and blood vessels as well as the interstitial space of surrounding tissue.

The compression of blood vessels leads to a reduced exchange of oxygen and nutrients that are essential for sustained tumour activity ^22^. This restricts the size of avascular tumours (to within 1 mm^3^) and induces hypoxic conditions within the tumour since the oxygen diffusion limit from the nearest blood vessels generally lies in the range 100–200 µm^23^. The compression stress associated with tumour growth can induce angiogenesis either by directly overexpressing vascular endothelial growth factor A (VEGFA) secretion or, indirectly, by blocking existing blood vessels to promote hypoxia and VEGFA secretion^16,24^. These collective factors, which include hypoxia, nutrient deprivation, and reactive oxygen species, are detrimental to cell survival. However, they produce a pressure that acts selectively on metastatically fit cells, initiating the process of metastasis^25^.

The interstitial fluid of almost all tissues consists of plasma filtrate that escapes from the blood capillaries and drains into the lymphatic vessels flowing through the interstitium^17^. Interstitial tissue fluid pressure (IFP) normally ranges between −2 and 0 mmHg, while tumour IFP can be 10 times higher, with values reported in humans as high as 60 mmHg^26^. This results from fluid accumulation in the interstitial spaces as a consequence of increased vascular permeability, blood vessel compression, and reduced lymphatic drainage^7^. Elevated IFP results in the transportation of pro-angiogenic factors from the tumour centre to its periphery in response to hypoxia, thus inducing peripheral tumour-lymph angiogenesis and peritumoural haemangiogenesis, subsequently leading to lymph-node metastasis^27^. This increase in tumour IFP limits the transport of systemically delivered therapies owing to the formation of a high-pressure gradient near the tumour periphery, opposing the intended direction of drug distribution from blood vessels^28^. Breast cancer cells cultured in a 3D collagen matrix under fluid-flow conditions representative of IFP, exhibit a decreased responsiveness to an anti-cancer drug treatment (doxorubicin)^29^. Smaller drug molecules diffuse more easily towards regions of increased fluid flow, while the efficacy of larger therapeutic strategies, such as immunotherapies, is reduced because of poor drug penetration^30^. A strong correlation between IFP and high microvascular density and metastasis to lymph nodes in human melanoma xenografts has also been reported^27^. Lowering the IFP in solid epithelial tumours by puncture-draining the central cystic area resulted in reduced tumour proliferation in mice^31^. More recently, real-time histologic imaging of human tissues revealed the structure of the fluid-filled interstitial space, leading to the possibility of direct sampling of interstitial fluid for diagnostics^32^. Interestingly, ongoing research suggests the existence of interconnected body-wide network of fluid-filled interstitial spaces across tissue and organ boundaries in humans, which may reveal new insights into cancer pathophysiology ^33^. Overall, the above observations indicate that the total stress generated by the fluid and non-fluid (solid stress) components of a tumour promotes a malignant environment and limits the delivery of most drugs.

## Detachment and invasion

Compared to normal cells, transformed epithelial cells display considerable differences in the intermediate filament profiles and cytoarchitecture. The transformed microrheology provides cells with a distinct advantage when undergoing intravasation. Many cancer cells undergo a hallmark epithelial-to-mesenchymal transition (EMT), which involves a change from a keratin- to a vimentin-based cytoskeleton^34^. Cells in a mesenchymal state are typically more motile than their epithelial counterparts, and undergo simultaneous morphological changes during an EMT, including elongation, changes to cellular polarity, and adhesion. This transition is shown to soften cancer cells in the head and neck to promote their migration in 3D spatially constrained environments^35^. However, it should be noted that this transition is not a binary switch that shunts cells from a fully epithelial to a fully mesenchymal extreme. Rather, it is a continuum that allows a rapid interconversion between the two traits, demonstrating a high phenotypic plasticity ^36^. It is well recognised that the microenvironment influences the transition between epithelial and mesenchymal states through signal transduction. For instance, exposing hepatocellular carcinoma to a hypoxic condition leads to the overexpression of HIF-1α (a key transcription factor that regulates a cell’s response to hypoxia) and induces a mesenchymal phenotype via the activation of SNAI1^37^. Similarly, the regulation of EMT by the substrate stiffness has been reported in many cancer types, both in vitro and in vivo^38,39^. Hence, in the presence of compliant substrates that usually suppress the spread and proliferation of normal cells, they support an extensive proliferation of transformed cells and exert abnormally high traction forces that can interfere with the cellular junctional integrity, compromising tissue polarity, fostering anchorage-free survival, and enhancing invasion^6^.

As a cancer cell begins to invade the surrounding parenchyma, several force-generating mechanisms come into play. The increase in cell contractility is reflected by an overexpression of the Rho protein and an increase in Rho GTPase activity, along with its downstream effectors. In addition, growth-factor-induced ERK activity, which regulates cytoskeletal tension, is elevated. It has been shown that epidermal growth factor receptors (EGFR) in tumours become activated by compressive stress, and that the ECM stiffness promotes the activation of the ERK pathway by utilising EGF ligands^40^. As a result, cancer cells generate protrusive processes known as invadopodia to digest, invade, and remodel the ECM through a coordinated action of several proteins binding to actin, such as Arp2/3, N-WASP^41^. Such local dynamic force generation in the extension-contraction cycles of cell protrusion facilitates highly localised actin polymerisation in invasive cancer cells, which then enables spatially focused proteolytic secretion^42^. This results in a rapid and non-reversible remodelling of the existing matrix and a secretion of a new densified matrix at the cell vicinity to support subsequent invasion^43^.

Despite being a hallmark, the EMT is neither necessary nor sufficient for metastasis. Certain cancer cells, known to retain many epithelial markers while not upregulating mesenchymal markers, can still metastasise. This can be explained by clonal cooperativity within the tumour itself, wherein a small percentage of EMT cells (aggressive subclones) enhances the invasiveness of non-EMT cells (non-aggressive subclones), leading the non-EMT cells to intravasate^44^. An alternative explanation is that cancer cells (here, squamous cell carcinoma, lung adenocarcinoma) can also employ stromal fibroblasts to remodel the ECM and pull carcinoma cells within the tracks created by the fibroblasts. This occurs via mechanically active cadherin adhesions formed between the two cell types^45^. Another interesting recent theory called the unjamming transition (UJT) considers the dense primary cancer cell mass as a solid-like jammed phase that transitions to a fluid-like unjammed state during cancer invasion while retaining its epithelial phenotype throughout^46^. Decreases in cell-cell adhesion and in ECM confinement have been seen to accelerate the fluidisation of breast cancer cells at the tumour periphery^47^. While some parallels can be drawn between the UJT and EMT theories in cancer invasion, the UJT largely holds true even when EMT does not.

The overproduction of ECM components around tumour clusters results in reduced ECM porosity, which hinders cancer cells penetrating through small pores. Thus, intracellular alterations in mechanical properties by the degree of cytoskeletal reorganisation and active mitochondrial localisation also determines the cellular transition from a non-aggressive to a malignant phenotype^48^. Amongst the cytoskeleton components, which include microtubules, vimentin intermediate, and actin filaments, the F-actin filaments are more resistant to deformation, followed by intermediate filaments, and with least contribution by microtubules^49^. Thus, the cytoskeleton must soften to enhance cancer-cell motility, facilitating the cell to be more stretchable, deformable, and easily contractible. One study segregated stiff (>700 Pa) and soft (<400 Pa) cells from a heterogenous tumourigenic cell population and observed that the soft cancer cells formed a tumour after injection in immunocompetent mice. In contrast, there was no tumour formation with the stiff cells^50^. The accumulation of migratory machinery components, including actin and active mitochondria, at the front of the cell has been shown to increase migration speed in highly confined spaces^51^. Intravital imaging revealed the adaptive actin dynamics in the invasion front and the invasion-guiding tissue structures before intravasation in vivo, which was previously technically challenging^52,53^. More malignant cells also possess greater contractility and intracellular viscosity. This is evident in a cancer spheroid invasion assay that shows a consistent increase in intracellular viscosity of spheroids embedded in a 3D matrix, wherein invasive strands of the spheroids have a more viscous cytoplasm than cells at the spheroid core^54^. Additionally, the plasma membrane in lung cancer cells displays increased membrane fluidity, correlating with the invasive potential and a poor prognosis^55^. Hence, the only cells capable of undergoing cytoskeleton rearrangement, or those that employ proteolytic cleavage mechanisms either directly or indirectly to remodel the ECM, can pass through spatial constraints and migrate into surrounding tissues.

## ECM remodelling

The extracellular matrix is a highly complex system that normally provides a niche for various cell types to proliferate, collaborate, differentiate, and apoptose^56^. The ECM comprises approximately 300 different proteins, 35 proteoglycans (PG), and 200 glycoproteins that perform many critical functions. Most of these functions have clear mechanical roles, including providing structural and mechanical integrity. Mechanically, within the ECM, collagen is known to resist tensile forces^57^. The glycoproteins (such as fibronectin, tenascin) crosslink the fibres in the ECM (such as collagen, elastin), thereby increasing its resistance to applied forces and modulating its elastic or plastic behaviour (i.e. allowing it to revert to its original form after the relaxation of an applied force (elastic) or remaining deformed (plastic) (Box 1))^19^. The composition of the ECM crosslinks and their molecular bond kinetics determine the viscoplastic properties of the ECM. They play a key role in tension propagation and stiffening in response to cell interactions^58^. These bonds are often heterogeneous in tissues (based on the secretion of crosslinks, important in many solid tumours), leading to complex local- and network-level features of the ECM in tumours. In addition, the resultant ECM bundles have variable micron-scale pore sizes through which a cell can migrate by adopting a wide array of mechanisms, as previously described^59^. During normal maintenance activities under physiological conditions, a tightly regulated balance exists between the production and degradation of fibres, PGs, and glycoproteins. However, during cancer progression, the matrix near most solid tumours is often stiffer than in normal tissue owing to increased collagen deposition. For instance, the stiffness of glioblastoma tissue is ~25 kPa, while normal brain tissue stiffness lies in the range 0.1–1 kPa^60^. Thus, alterations in the ECM stiffness, along with changes in its composition, spatial organisation, and topography, influence the progression of cancer dissemination as well as the therapeutic response^61^. Cell-ECM interactions are often identified as a hallmark for many physiological and pathological conditions involving cell migration.

Amongst the ECM scaffolding proteins, fibrillar collagen is abundant and identified as a major contributor to the ECM stiffness. Extensive studies have been reported on cancer cells modifying the mechanical properties (pore size, density, stiffness, viscoelasticity) of collagen using complex inter- and intracellular signalling pathways^5,62^. In an in vitro study, the contractile forces originating from the expansion of mouse colon cancer spheroids embedded in a biomimetic collagen matrix, deformed the ECM, generating tensile radial forces within the matrix, which in turn reduced invasion upon relaxation^63^. This observation highlights the existence of unbalanced forces between a tumour and the ECM, which drives a physical relation between the ECM tensile state and cancer cell invasion. In an attempt to balance the forces, cancer cells adapt to changes in the surrounding tissue by altering their mechanical properties to prolong their survival inside the ECM. For instance, an increase in the matrix tension directly impacts the formation and response of the cytoskeleton by inducing EMT^64^. In addition, increased collagen-VI levels in cancer tissues triggered the AKT pathway in MCF-7 breast-cancer cells, causing the upregulation of proliferation genes and increased angiogenesis, indicating that collagen composition can direct cell fate in tumours^65^. Three signatures define matrix topography, denoted as tumour-associated collagen signatures (TACS). The first is wavy collagen, which is like a typical mammary gland but with a higher density in the tumour vicinity. The second and third can be characterised by straightened and aligned collagen fibres oriented parallel and perpendicular to the tumour edge, respectively^56^. The alignment of collagen fibres facilitates migration and aids a cancer cell’s journey towards blood and lymphatic vessels. Mesoscale features of collagen fibres (i.e. wavy and thickened fibrils) have also been mimicked in vitro, displaying changes in the phenotypes and migratory ability of both tumour and normal cells^66,67^. In a breast cancer cell line, the ATP:ADP ratio, marker of energy consumption, increases in denser collagen matrices, where migration is impaired, and the ratio decreases in aligned collagen matrices^68^. More recent work by the same group highlights that these cells prefer to migrate in the direction of the tension (in collagen fibres), rather than along the collagen alignment, to minimise energy cost^69^. This effect points to a new intratumoural signature involving the selection of an energy-efficient migration path.

Most cancer cells overexpress collagen-modifying proteins to remodel the ECM, via mechanisms such as matrix metalloproteinase (MMP)-dependent cleavage and lysyl oxidase (LOX)-mediated crosslinking of collagen^70^. The crosslinking mediated by LOX results in enhanced tissue stiffness and FAK/SRC signal activation in both in vitro and in vivo models of colorectal cancer^71^. In some cases of lung cancer, downregulation of miR-29a, a small non-coding RNA molecule, causes an upregulation of LOX, resulting in the stiffening of the surrounding ECM. This suggests that cancer cells can also utilise non-coding RNA to stiffen the ECM^72^. On the other hand, MMPs are associated with the enzymatic degradation of various ECM proteins (fibronectin, collagen, PGs) to aid cell invasion^73^. However, in one study, the rate of collagen degradation via enzymatic cleavage decreased considerably when the collagen fibres were mechanically deformed owing to the restricted access of enzymes to the cleavage sites on the collagen molecule^74^. Thus, cancer-cell invasion may require a combination of ECM degradation proteins along with high cell traction forces for invasion and metastasis to be effective. Paradoxically, tumour cells increase the tissue stiffness (e.g. LOX), which is believed to physically restrict invadopodia extensions, impeding cell movement^75^. Later, they must partially degrade (e.g. through MMPs) or remodel it, which mitigates the importance of tissue stiffness. This could be explained in terms of the simple force balance required while walking, i.e. a linearised and stiffened ECM provides an equal and opposite force that cells can exert (through traction) to migrate by increased integrin-mediated focal adhesions, which is rather difficult in soft tissues. In other cases, cancer cells can also adopt a plastic mode of migration, involving a transition to an amoeboid state that utilises protease- and anchorage-independent migration to progress through the ECM. An interfibrillar pore cross-sectional area as small as 7 µm^2^ was found to constitute a sufficient physical barrier for arresting tumour-cell migration (at about 10% of the nuclear cross-sectional area); the cells hence rely heavily on MMP-dependent cleavage to enlarge the matrix pores and on integrin- and actomyosin-dependent force generation to jointly propel the nucleus^76^. Hence, it can be said that cancer cells may be primed for metastasis by modifying their physical extracellular environment as nicely reviewed by Paul et al.^77^.

Mechanistically, a stiff ECM increases intracellular tension through actomyosin contractility, alters cell metabolism^78^, and encourages cell proliferation^7^. This effect in turn further stiffens the surrounding tissue through the activation of integrin-mediated focal adhesions^79^. Integrins are mechanosensors that connect the ECM to the intracellular actin cytoskeleton through several mechanosensitive adaptor proteins, such as talin, vinculin, and α-actinin present inside the cell. Cancer cells anchor the ECM through integrin-mediated binding and generate traction forces against the ECM adhesion because of cellular contraction facilitated by myosin, to move forward^80^. Whilst increasing focal adhesion (FA) is important for ECM attachment, a faster FA disassembly is another essential factor for effective migration^81^. Thus, cancer cells that form weak adhesions and have a faster FA synthesis turnover are more aggressive and metastatic compared to their strongly adhering counterparts, signifying the existence of an intratumoural heterogeneity within a given cancer cell line^81^.

Through extensive extracellular remodelling, cancer cells and neighbouring stromal cells disturb the tensional homoeostasis and develop a tumorigenic environment that favours cancer-cell survival, immune evasion, and migration. A stiffer ECM can also produce molecular as well as epigenetic changes within a cell, including differential levels of microRNA, which can be correlated with tumour aggression^82^. Living tissues and ECMs do not exhibit linear elasticity. Rather, they display a more complex combination of mechanical actions, such as viscoelasticity, plasticity, nonlinear elasticity, and poroelasticity, which comprises time- and rate-dependent behaviours^83^. Such mechanical patterns are gaining attention, and forthcoming research will likely provide further understanding of cell behaviour. Nevertheless, tissue stiffness remains a widely recognised originator of a cascade of manifold changes within a cell as well as its neighbouring cells and ECM remodelling. Targeting tissue stiffness and its modulators may create interesting targets for novel therapies as well as enabling early diagnosis.

Box 1 definition of some common mechanobiology concepts *Stress*: any environmental pressure that elicits a response from an object is called stress. It can be expressed as applied force per unit area, expressed in Pascal (Pa) or newton per square metre (N/m^2^).

*Solid stress*: the stress exerted by a tissue’s solid constituents that accumulate during tumour growth within the solid structural components (e.g., the ECM, tumour).

*Shear stress*: the stress tending to cause deformation when a surface is displaced tangentially.

*Fluid shear stress*: the shear stress caused by the moving layers of a laminar fluid flow as a consequence of their internal frictional force.

*Interstitial flow*: the fluid flow arising from the leakiness of the blood capillaries that supply essential nutrients to cells, draining into lymphatic vessels.

*Interstitial fluid pressure*: the interstitial hydrostatic pressure that is frequently elevated in solid tumours owing to blood-vessel leakiness and impaired lymphatic drainage.

*Stiffness*: a measure of a material’s resistance to deformation under mechanical stress. Its units are similar to stress, i.e. Pascal (Pa) or Newton per square metre (N/m^2^).

*Young’s elastic modulus*: an intrinsic solid-material property that quantifies its stiffness. It is defined as the ratio of the stress acting on a substance to the resulting strain (deformation).

*Viscoelasticity*: a property of a material (here, a cell) that exhibits both viscous (fluid-like) and elastic (solid-like) characteristics during deformation.

*Plasticity*: the ability of a cell to modify its physiological characteristics to take on a new phenotype when under myriad causes of stress.

*Note*: Solid stress and stiffness are discrete biomechanical abnormalities. Stiffness is a property of the material that resists its deformation in response to an externally applied force whereas, solid stress is the force applied on a surface that causes either the expansion (due to tension) or compaction (due to compression) of a material. Solid stress in breast tumour models increases with increasing tumour size despite similar stiffness values^254^.

## Heterogeneous cell interactions

Tumours are well known to display enhanced stiffness relative to nearby uninvolved tissue. This fact is made evident by palpation, a diagnostic approach used for soft tissues like breast. This observation forms the basis for recent high-resolution detection techniques for small lesions, involving magnetic resonance elastography or ultrasound^84^. Tumour cells themselves are unlikely cause of increased stiffness, which seems to result rather from the combined effort of stromal cells that synthesise the matrix. This effect substantially affects the different cell types present in the TME and facilitates the immune response in the tumour stroma. As a primary tumour increases in size and volume, it promotes oncogenic signals to induce inflammation. Consequently, the primary organ becomes inflamed with immune cells such as macrophages, neutrophils, and mast cells, which secrete reactive oxygen species (e.g. nitric oxide) that play a role in tumour angiogenesis due to increased IFP^25,85^. The large number of infiltrating macrophages in human breast tumour biopsies correlates positively with the ECM stiffness and cellular TGFβ signalling at the invasive front, regardless of the subtype^86^.

Despite the perceived inflammation at the tumour site, this accumulation of different cell types may constitute an immunosuppressive environment and lead to the inactivation of T cells (the presence of which signifies a good prognosis). An increased collagen density modulates the reduced efficiency of macrophages for attracting cytotoxic T cells and inhibits T-cell proliferation, compared to macrophages cultured in lower-density collagen^87^. Similarly, the activity of T cells is significantly reduced in highly dense collagen matrices and, in some cases, their infiltration is completely hindered (so-called 'cold tumours')^88^. The clinical testing of an antagonist endothelin B receptor (bosentan) to improve the homing of T cells to tumour blood-vessel walls was not approved, possibly because of compressed vessels that prevent perfusion and T-cell attachment. Thus, increased T-cell trafficking in vascular endothelium in a preclinical setup has been observed when applying measures to normalise the TME by reducing the tumour stiffness to decrease collagen and, to decompress the vessels by adding endothelin A receptor inhibitor^89^.

Mesenchymal stromal cells (fibroblasts, adipocytes) constitute a major group of the TME. They become activated by mechanical stresses and perform mechanical roles in promoting tumour progression besides secreting soluble factors. Cancer-associated fibroblasts (CAFs) are an activated form of fibroblasts, often nicknamed the 'cockroaches of the human body' for their ability to survive severe stress that are often lethal to all other cells^90^. Another nickname is 'engineers of the ECM', as they can synthesise and remodel the interstitial matrix^91^ by depositing ECM components such as fibronectin^92^ and collagen. In vitro experiments on lung and pancreatic cell lines show cancer cell migration on elongated protrusions of fibroblasts, similar to trains on railway tracks, through integrin α5β1-mediated binding to fibronectin on a fibroblast surface^93^. Several studies have highlighted that CAFs utilise both proteolytic (via MMPs^94^) and force-dependent (via Rho-ROCK-Myosin II^95^) activity to modify the ECM. In some cases, matrix remodelling is independent of MMP secretion. Instead, CAFs drastically pull, stretch, and soften the basement membrane, generating gaps for cancer cell migration^96^. Although CAFs are known to promote tumour invasion^97^, there is also evidence that they can hinder invasion by forming a physical barrier around the tumour^98^. Hence, the classification of the heterogeneous population of this critical cell type will be essential for advancing our understanding of CAFs and for designing cautious stromal-targeting strategies^99^. Another cell type in the stroma, called adipocytes associated with obesity, also promotes invasion through both direct cell contact with tumour cells and MMP-mediated ECM remodelling^100^. Thus, highlighting the importance of more focused studies exploring the physical cell-cell heterogeneous interactions maybe useful to achieve a better understanding of cancer invasion.

A subpopulation of cancer cells called cancer stem cells (CSC), has also received considerable attention recently. The intrinsic plasticity of these cells may be able to regenerate an entire tumour and increase drug resistance despite their small percentage within the overall cancer cell population^101^. Ovarian cancer stem-like cells show an increased ability to deform by up to 72% in comparison with non-malignant early-stage ovarian cancer cells, constituting a possible biomarker for CSC screening^102^. There is also growing evidence that the mechanical stress induced by a stiffened matrix is sensed and transduced by mechano-sensors to induce pathways for CSC survival, renewal, and differentiation^101^. At the cellular level, the actin-dependent control of YAP/TAZ and mechanosensitive transcriptional regulators are associated with increased CSC-like properties^103^. Mechanical stress on the primary tumour defines their proliferative capacity through YAP and TAZ mechanoregulation. YAP/TAZ activity have been shown to serve as a mechanical checkpoint that controls contact inhibition of proliferation^104^. Overall, a detailed mechanistic understanding of the cellular TME and its physical interactions with other cells, especially via the ECM, is essential for developing novel targeted therapies.

## Mechanobiology of early dissemination—intravasation

The major source of dissemination of cancer cells is through the circulatory system, either directly via blood vessels (hematogenous) or through lymph vessels (lymphatic). Experiments have revealed that the intravasation of (breast and pancreatic) cancer cells into lymphatic vessels is more probable than into blood vessels. This is because (i) lymphatic vessels do not have dense inter-endothelial junctions, and (ii) the circulation in lymph is slower than in the blood vessels, reducing the coincident shear stress and increasing the probability of survival^105^. Eventually, CTCs in the lymphatic system enter lymph nodes before draining into the blood circulation, a potential explanation of why lymph nodes are common secondary metastatic sites. Before entering the circulation, cancer cells must interact with endothelial cells to facilitate the opening of endothelial cell-cell junctions, to squeeze through the junctions, as well as lose anchorage dependency for cell-cycle progression and survival^106^. The cells’ escape is promoted by the outward interstitial fluid flow that results from the pressure gradient created by a high IFP within the tumour^17^. Cells capable of surviving these stresses have a high metastatic potential. Non-metastatic carcinoma cells become fragmented during intravasation, while metastatic tumour cells cross the basement membrane and the endothelium as intact cells^107^.

The tumour stroma dynamically influences and promotes intravasation. Both perivascular macrophages and CAFs instruct cancer cells to intravasate by secreting EGF, TNFα, TGFβ, and CXCL12^108^, which polarise cancer cells towards the blood vessels^109^. However, little is known about the physical interactions between stromal cells and the cancer cells supporting intravasation. Since intravasation depends primarily on the access of tumour cells to blood vessels, intensive research has brought to light different mechanisms employed by tumour cells to enter circulation, in addition to the well-recognised angiogenesis phenomenon. As cancer cells approach endothelial cells, β-catenin-mediated upregulation of N-cadherin (a mechanosensor) in the endothelium promotes the physical attachment of cancer cells to the vasculature by virtue of the increased matrix stiffness^110^. Next, myosin-II contraction in endothelial cells likely increases the gap size between the adjacent junctions to facilitate tumour cell transmigration^111^. However, the gap may not be sufficiently large for cells to avoid the distortion of the nucleus, whose typical higher stiffness makes it the greatest impediment to migration. Thus, the cancer cell nuclear shape and nuclear strain are crucial determinants of transendothelial migration while squeezing through the endothelial vasculature as the nucleus modulates gene expression and cell differentiation^112^. The nucleus of metastatic tumour cells is observably softer, which along with high traction force exerted at the integrin adhesion sites, enables metastatic cells to navigate through a constraining environment^113,114^. Low levels of lamin A (a nucleoskeletal protein) have been identified to support confined cell migration in human breast tumours. This is associated with decreased chances of disease-free survival^115^. Hence, cancer cells could overcome these hurdles and avoid potential cell death during transmigration via nuclear deformability coupled with extensive DNA damage repair mechanisms^116^.

From the broader perspective of growth, invasion from primary tumour, and intravasation, the mechanical hallmark of solid tumour growth can be defined in terms of increased solid stress, enhanced interstitial fluid pressure, outward interstitial fluid velocity, massive tissue stiffening, and increased vascular permeability. At the tissue level, the deposition of ECM components accounts for the matrix stiffness, outlining signatures such as increased collagen content and density, its enhanced crosslinking, and hyaluronan swelling because of IFP. Cancer cells sense changes in the ECM and cell crowding and upregulate mechanotransduction pathways including, Rho-ROCK, ERK signalling, and the localisation of mechanosensitive transcription factors (e.g. YAP/TAZ) to the nucleus to regulate gene transcription in favour of cell survival. Similarly, cytoskeletal remodelling is also transmitted to the nucleoskeleton via LINC (linker of nucleoskeleton and cytoskeleton) protein complexes, which aids nuclear deformation and cell migration through the ECM and transendothelial migration. This is important as migration through a confined space challenges the integrity of the nuclear envelope and the genetic material, indirectly inducing genetic heterogeneity. An efficient repair mechanism would thus be required for survival^117^. In addition, the aggressiveness of cancer cells can be witnessed by mechanical signatures such as a low nuclear volume, increased intracellular viscosity, more focal adhesions (integrin clustering) but faster focal adhesion disassembly, high actomyosin contractility, low cell stiffness^118^, high traction forces exerted on the ECM, cell membrane fluidity, and altered cellular energy regulation (ATP:ADP ratio). Reduced ECM confinement and cell-cell adhesion aid the unjamming transition (UJT) of cancer cells from a dense primary tumour mass. Furthermore, following EMT, cancer cells may also prepare to shift from a mesenchymal (anchorage-dependent) to an amoeboid (anchorage-independent) mode of migration, commonly known as MAT for single cells. In the case of collective migration, the presence of a heterogenous phenotype in collective cells enhances the invasion potential and can also be a potent signature for invasion^119^. Interestingly, the process of cancer cell dissemination, previously considered to be a late-stage event, can in fact begin at an early stage of tumour growth hence, a deeper understanding and validation of these signatures is crucial for early diagnosis and prognosis^120^.

## Mechanobiology of the intravascular journey of CTCs

Following the entry of cancer cells into the vasculature, even primary tumour resection is insufficient to stem the systemic spread of cancer. Generally, single cancer cells circulate individually as CTCs or, in rare cases, as an aggregate-CTC cluster^121^ with the latter featuring a higher (23- to 50-fold) metastatic potential^122^. Circulating cells are exposed to the harsh environment of circulation caused by haemodynamic forces, immunological stress, and collisions with other cells circulating in the blood, as well as with the endothelial vessel wall lining^123^. All these stresses challenge cell survival. Consequently, cancer cells reprogramme their anchorage dependency or cell-matrix interactions by transitioning from adherence to suspension. Studies involving breast-cancer patients and mouse models revealed an upregulation of hematopoietic transcriptional regulators, which can impair cell-ECM adhesive properties^124,125^. The significance of the anchorage-independent transition in cells post-intravasation is not clearly understood and awaits further research. Only a few cells manage to survive in the bloodstream^123^. For instance, in metastatic breast and prostate cancer patients, the presence of 5 or more CTCs/7.5 ml of whole blood implies a significantly lower median progression-free and overall survival rate, compared to patients with <5 CTCs/7.5 ml blood^126^. The trajectory of CTCs in the circulatory system is influenced by various physical parameters, such as the blood-flow pattern, the blood-vessel diameter, and the dynamic interplay between shear flow and cell-cell adhesion that contributes to the intravascular arrest of cell movement in large vessels. Thus, to survive, metastatic CTCs must respond to changes in shear stress during circulation and before extravasation.

Shear stress arises at the interface of two fluids (blood in the present case) moving with different velocities. This effect can be understood as resulting from the viscosity-dependent internal friction force due to fluid flow, which is maximum at the vessel walls and minimum at the vessel centre. Hence, a high fluid shear stress at the walls prevents CTCs from settling and can potentially cause cell-cycle arrest and even cell death, forcing the cells to attain a state of plasticity. For instance, shear stress has been shown to induce EMT in CTCs, promoting a mesenchymal-cell-like potential in the systemic circulation of human breast tumour cells by downregulation of the ERK pathway^127^. Another study showed the induction of a cancer stem-cell-like phenotype in circulating MCF-7 cell lines without much change in its EMT expression, acting as a measure to survive physiological fluid shear stress in vitro^128^. This suggests that a dynamic change in the CTC cell state permeates throughout the circulation. This interchange in their cell state can be seen with the treatment response and disease progression in cancer patients treated with different therapies^129^.

Recently, breast cancer cells in patient blood samples showed a significantly different deformation pattern and higher shape restoration compared to the healthy peripheral blood cells during the mechanical characterisation using an optical stretcher, suggesting a potential mechanotype of CTCs^130^. Other cells, such as platelets, neutrophils, and macrophages in the bloodstream interact with CTCs and prevent them from rapid attack by NK cells^131^. Interaction with these cells provides a multi-faceted advantage by shielding the surface of cancer cells from shear forces, NK-cell-mediated lysis, and increases the probability of binding to capillary beds to facilitate extravasation at distal site^132^. Although a CTC cluster’s size and multicellularity make them more resistant to apoptosis, they can easily be physically trapped in the vessel lumina^122^, in spite of the individual cells in the CTC cluster being significantly smaller than single CTCs^133^. In addition, a CTC cluster shows increased stemness owing to specific changes in DNA methylation which do not occur in CTCs^134^. In some cases, a CTC cluster can also comprise other cells, including stromal (fibroblasts^135^) and immune cells (leucocytes^136^) that provide an additive survival advantage to the cancer cells by protection from apoptosis, endothelial-wall attachment, and early proliferation in the distant organ^137^. Hence, the interplay between intercellular adhesion in a CTC cluster enables cells to withstand a high shear stress and is becoming an emerging mechanical signature that potentiates metastasis^138^.

## Mechanobiology of intravascular arrest and extravasation

After escaping from shear-mediated or immunological destruction post entering the bloodstream, cancer cells adhere to a distant organ transiently or stably and begin extravasating to colonise the target organ. Metastatic onset depends on the successful arrest of intravascular CTCs before extravasation. Two main responses (which are not mutually exclusive) precede extravasation: physical occlusion (or entrapment) in microvessels (or capillaries), and active adhesion of CTCs to the vessel wall. For physical occlusion, if a CTC or CTC cluster enters a vessel with narrower diameter than itself, its arrest can then occur via physical entrapment owing to size restriction^139^. Although CTC clusters were previously believed to arrest immediately within such capillary-sized vessels, there have been reports of cells in CTC clusters reorganising their intercellular adhesions to form a single-file chain of cells to traverse through constricted capillaries^140^. This provides CTC clusters with a remarkable metastatic potential to disseminate to distant organs. While some organs are more susceptible than others to initiate secondary growth, organs with small capillaries are major sites of CTC arrest and adhesion. This is because, amongst microvessels, capillaries have a small luminal diameter (about 3–8 µm), low shear stress, and they cover a large surface area which can mostly trap clusters (mean diameter 12–20 µm). These features increase the probability of physical occlusion in capillary beds^141^.

The active binding of cancer cells to blood-vessel walls is influenced by the interplay between cellular velocity and adhesion. The human circulatory system presents drastically different blood flow patterns, with the arteries carrying pulsatile flow (i.e. with periodic variation) and veins carrying laminar flow (smooth, non-turbulent). Laminar fluid flow produces minimal fluid velocity at vessel walls, which, along with collisions with other cells in the blood, causes CTC margination towards the walls^139^. Shear stress also influences the translational and rotational motions of CTCs, governing the orientation and time constant related to receptor-ligand interactions resulting in adhesion^139^. Experimental evidence on zebrafish embryos reveals blood-flow profiles (400–600 µm/s) that are optimal for an efficient integrin-mediated adhesion force (>80 pN) between CTCs and endothelial cells^142^. In addition, overexpression of bulky glycoproteins has been identified in CTC patient samples that could mechanically enhance cell-surface receptor activity^143^. After adherence, CTCs must withstand a significant shear force to strengthen cell-cell adhesions to migrate and extravasate, the success of which also depends on the flow rate, as elevated flow profiles trigger endothelial remodelling^7^. Shear flow may also activate specific signalling pathways and transcriptional programs in tumour cells, leading to a dramatic reorganisation of the cytoskeleton and the adhesive machinery^144^.

Once trapped cells adhere to the vessel walls, they either undergo transendothelial migration (TEM) and cross the basement membrane or form an endothelial dome formation pocketing around arrested CTCs to colonise the secondary site, regulated by fluid shear stress^142^. Initially, trapped cells interact with surrounding endothelial cells through integrin or cytokine-mediated signalling^145^. However, certain melanoma cells that lack surface integrins or sialylated molecules bind to neutrophils through intercellular adhesion, and these in turn bind to endothelial cells via β2 integrin^136^. This enhanced cancer-cell adhesion to endothelial cells via tumour-leucocyte aggregation, is in part regulated by the hydrodynamic shear rate^136^. Another mechanism by which haemodynamic shear stress promotes extravasation in both in vivo and in vitro models, is the upregulation of ROS production levels in tumour cells with fluid shear stress (5-15 dyne/cm^2^) stimulation, activating ERK signalling pathway and promoting TEM^146^. For TEM, cancer cells extend invadopodia-like protrusions through the endothelial junctions into the extravascular stroma preceding extravasation, whose inhibition is shown to abrogate extravasation^147^. Another study found that these actin-rich protrusions generate complex push-pull forces to drive TEM, while the success of cancer cell transmigration also relies on endothelium-generated forces to facilitate gap formation^148^. This highlights the dynamic nature of endothelial gap formation, which can also be regulated by the fluid flow just as it induces polarity in endothelial cells^149^.

Overall, the major contributors to successful extravasation are increased cell survival, cell arrest, and adhesion to vessel walls. Suspended tumour cells that manage to survive haemodynamic shear stress often get arrested or actively adhere to regions of reduced blood-flow dynamics, signifying a potential mechanical signature of arrest. It is speculated that arrested or adhered tumour cells upregulate mesenchymal markers to increase the spreading area for attachment, and form invadopodia in the early phase of circulation itself. Most CTC transit within blood vessels is flow-driven and does not involve cell motility. Thus, the process of circulation witnesses a relatively reduced mesenchymal phenotype whereas, during cancer cell arrest, the pericyte-like spreading of cancer cells signifies that fluid shear stress may promote EMT. This variation of cell behaviour within the circulation for transit to secondary sites awaits detailed study, with clinical validation, to provide a possible explanation of CTC chemoresistance. Chemoresistance may also be explained by heterogeneous cells existing in close proximity within a CTC cluster, overcoming hindrances synergistically. Similar to intravasation, an extravasation event also requires the activation of the YAP/TAZ transcription, regulating motility and the cell cycle under high wall shear-stress conditions^150^. In addition, the upregulation of bulky glycoproteins on a CTC surface to physically interact with endothelial walls and increased cellular and nuclear deformability (to enable the passage of cancer cells squeezing through a vascular wall) are required in favour of extravasation. Also, cancer cells have a more disorganised and less filamentous cytoskeletal network with reduced actomyosin activity, when compared to healthy cells, to enable TEM. Hence, another signature of suspended tumour cell could be a reduction in F-actin assembly and cell softening that would give cancer cells a survival advantage^151^. It can be concluded that the delivery and intravascular arrest of cancer cells is primarily a flow-driven process that involves withstanding shear stress, mediating physical interactions, and cellular alterations to facilitate extravasation.

## Mechanobiology of secondary tumour formation

The CTCs that cross the final physical (endothelial junction and BM) barrier are referred to as disseminated tumour cells (DTCs). They enter an alien microenvironment characterised by mechanical forces that potentially differ from those of the primary tumour site. Depending on the microenvironment, DTCs may remain quiescent in a non-permissive environment, or they could prepare the environment prior to extravasation for active colonisation. Dormant DTCs show striking similarities to CSC-like cells while transitioning between quiescent and reactivation states^152^. A common pathway employed by DTCs for metastatic colonisation in multiple organs is pericyte-like spreading, where tumour cells localise in a vascular co-option pattern^153^. Such metastatic derivatives of lung, breast, and colon cancer cells were observed to be spreading across the host tissue capillary, competing with pericytes for interaction with the endothelial basal lamina and replacing its position by activating YAP and MRTF, mechanotransduction effectors^153^. Interestingly, intravital microscopy of mouse colorectal cells colonising the liver through confined spaces showed that cancer cells were active and motile following extravasation and the inhibition of cell motility reduced metastatic burden before they could form micro-metastases^154^. Alternatively, a report utilising EMT lineage tracing assays concluded that most secondary metastases are caused by cancer cells with the epithelial phenotype that did not undergo EMT, bypassing the well-recognised EMT/MET transformation that might not be required for dissemination^155^. It could thus be speculated that the formation of secondary metastasis from CTC clusters is a synergistic sequence of events in which mesenchymal cells (either CAFs or EMT positive cells) cooperate with non-EMT cells to enter and exit circulation to colonise while preserving the high proliferative ability of epithelial cells.

Recent findings revealed that certain microenvironments develop a pre-metastatic niche that initiates CTC arrival, a crucial step in metastatic tumour formation^156^. Several factors secreted by tumours are released into the blood, such as cytokines, chemokines, growth factors, MMPs, tumour DNA, and extracellular vesicles that travel directly through the systemic circulation to colonise distant organs, even before the CTCs arrive^157^. For instance, animal tumour models have shown an increase in vascular permeability at pre-metastatic niches by upregulating C-C chemokine receptor type-2 expression^158^. Vascular leakiness accompanied by changes in neighbouring resident cells such as fibroblasts, and non-resident cell recruitment such as bone marrow-derived cells (BMDC), subsequently attract CTCs^159^. Also, LOXL2 secreted by hypoxic head and neck cancer stimulates pre-metastatic niche formation and drives local invasion of non-hypoxic head and neck cancer cells^160^. The resident fibroblasts in this case, produced fibronectin, a new ECM component that can further form a permissive niche^160^. Moreover, tumour-derived exosomes (30–150 nm in size) comprise distinct integrin patterns that are taken up by organ-specific cells to prepare a pre-metastatic niche^161^. These factors are novel targets for early cancer detection or longitudinal tracking of anti-cancer response by utilising liquid biopsies^162^.

**Fig. 3:**
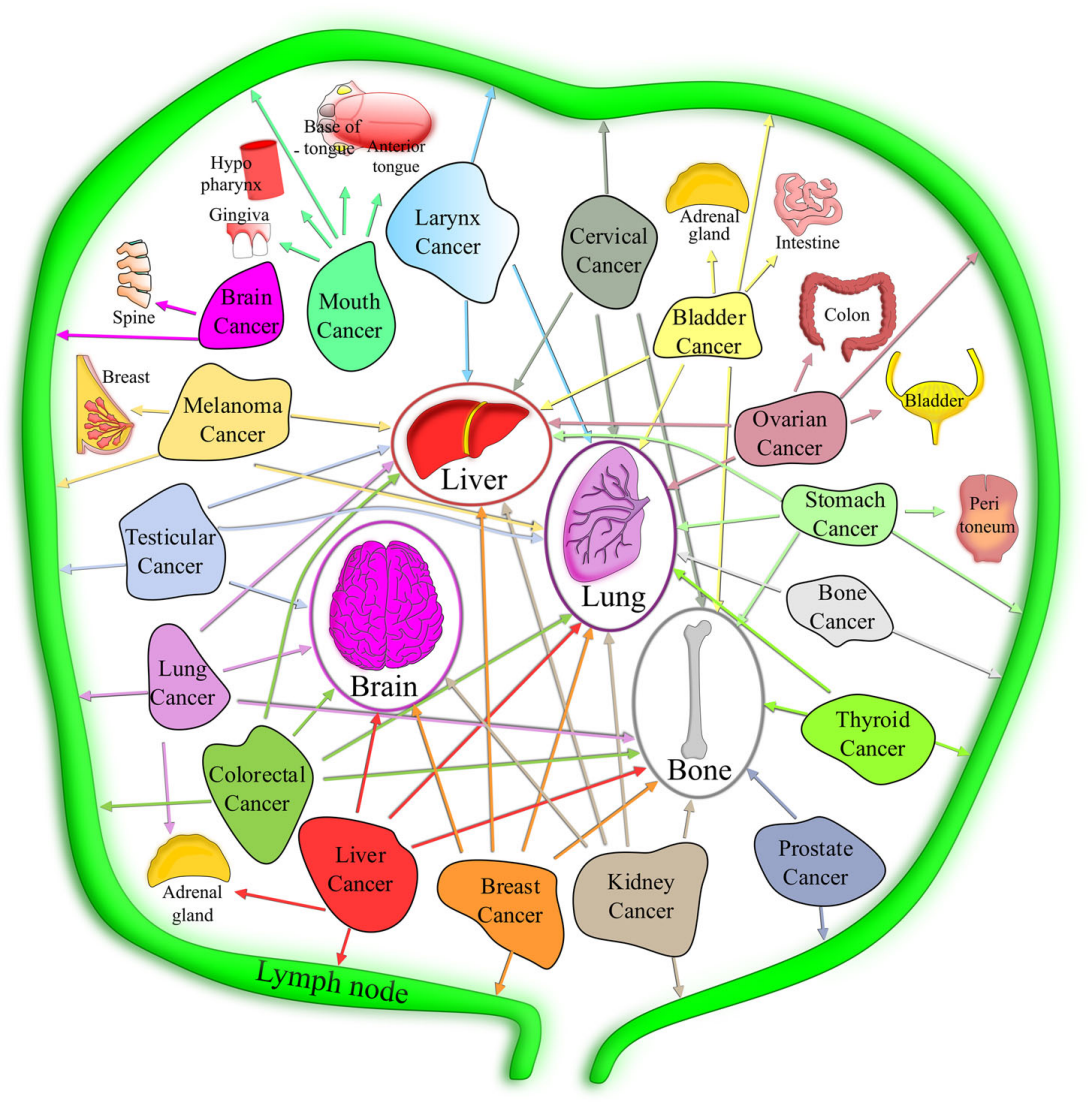
Common primary and secondary organs in cancer metastasis. Map representing the potential metastatic spread from some primary cancer sites to their main secondary sites (shown by arrows).

Many theories have been proposed to explain secondary metastatic colonisation (organ tropism). The very first theory defined was Paget’s 'seed and soil' hypothesis, whereby metastasising cancer cells are like seeds that can grow in a proper soil^163^, emphasising that the localisation of the seeds is not arbitrarily chosen. A subsequent alternative theory was Ewing’s 'mechanical' hypothesis, whereby the vascular system anatomy determines the site for metastatic dissemination, with regions of optimal flow patterns influencing adhesion and extravasation^164^. Some secondary metastatic sites for primary cancer were later confirmed experimentally^165^. This theory could explain why CTCs frequently metastasise to lung, bone, or liver owing to a bed of capillary-like vasculature (Fig. 3) and not to certain other organs as CTCs fail to reach them. However, active research continues to investigate the puzzle of the fate of cells and the reasons for factors influencing the process, once CTCs are guided to specific organs. Interestingly, the overall stiffness of the secondary organ is not a factor for determining a secondary site. For example, breast cancer cells can metastasise to the brain, lung (soft), and bone (stiff) tissues. However, organ stiffness can influence cell survival post-extravasation if the primary and secondary tissue stiffnesses differ. For instance, pancreatic cancer cells develop mechanical memory as a means to adapt to substrates of varying stiffness through YAP and mir-21 memory keepers^166^. The cells generate differential forces and display a distinct response to treatment in the cases of different secondary substrate stiffnesses, despite being cultured in the same past matrix stiffness^166^. Another possible explanation for this wide spectrum of substrate specificity could be heterogeneity within the tumour itself. For instance, MDA-MB231 breast cancer cells harbour different single-cell populations that respond to different extracellular matrix rigidities and potentially have different biomechanical properties, indicating different target-tissue specificities^167^. In addition, other tissue mechanical properties, such as viscoelasticity and poroelasticity, compound the complexity for deciphering the impact on tumour growth. Active research on this subject continues. Overall, fluid-flow profiles and vessel geometries in an organ determine the secondary sites, and metastatic cells are increasingly compliant as they transition to a lower stiffness relative to normal cells. These properties can be classified as prominent mechanical determinants that establish metastatic cancer. Post-survival, the vicious cycle of cancer invasion similar to primary tumour growth by mechanically challenging the microenvironment begins.

## Mechanical measurements in cancer

**Fig. 4:**
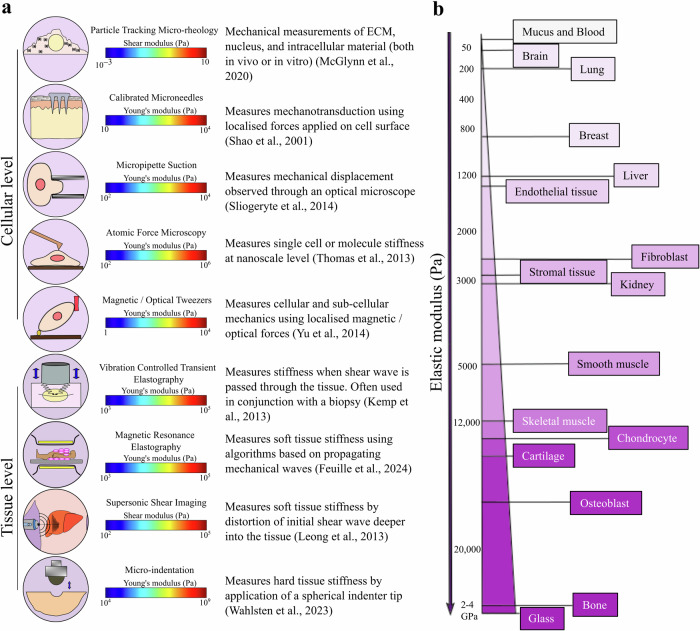
Mechanical measurements. **a** Techniques used to measure stiffness at the cellular and tissue levels^169–173,177–180^. **b** Range of Elastic modulus for different cells and tissues^24,236–239^.

A sophisticated suite of technologies has emerged over the last two decades, with the primary objective of quantifying cell and tissue mechanical properties, such as Young’s modulus, Poisson’s ratio^168^, and viscoelastic parameters (Fig. 4). The methods include measuring the rheological properties and mechanical responses of the whole cell to, e.g. micropipette aspiration^169^, microneedles^170^, magnetic tweezers^171^, traction force microscopy (TFM), atomic force microscopy (AFM)^172^, particle-tracking microrheometry^173^ and many more as reviewed by Hao et al.^174^. Some of these methods can be applied on the subcellular and whole-cell scales; for example, AFM has been used extensively at both low and high resolutions^175^. Quantitative phase imaging (QPI), a recent technique for measuring biomass fluctuations in cells, provides a probe- and contact-free approach for quantifying changes in cell viscoelasticity^176^.

Even in apparently static tissues, cells continuously experience a multitude of mechanical forces and, as a result, deliberately exert mechanical force on their environment^6^. Various methods exist for characterising mechanical tissue properties, such as magnetic resonance elastography (MRE)^177^, shear wave elastography (SWE)^178^, micro-computed tomography (µCT), quantitative computed tomography (QCT), vibration-controlled transient elastography^179^, micro-indentation^180^, and more as reviewed by Rao et al.^181^. Most of these techniques utilise imaging concurrently with mechanical loading to measure tissue biomechanical properties. One type of optical elastography, Brillouin microscopy, has recently emerged as a non-destructive, label- and contact-free method for measuring the viscoelastic properties of biological samples (in both intact cells and tissue samples) with a resolution limited by diffraction in 3D^182^. Despite progress in quantifying biomechanical parameters, acquiring new tools and techniques for effectively estimating force generation arising from IFP and solid stress within a tumour remains a challenge. It is important to continue developing new and concrete instruments for estimating mechanical factors.

## Challenges in determining mechanical forces

One major research challenge regarding the mechanical determinants of metastasis is the lack of standardised stiffness measurements. Stiffness is the most identified mechanical characteristic while models for the spatiotemporal estimation of other mechanical properties remain in their infancy. Reported mechanical properties vary significantly between the cellular and tissue levels, which requires care when deciding which measurement technique to use. At the cellular scale, cell differentiation and migration are regulated by viscoelastic properties. They determine the cellular response to physical forces and to their environment, while mechanical tissue properties dominate morphogenesis and multicellular organisation^182^.

In a clinical setting, non-invasive methods for measuring tissue stiffness are quicker, cheaper, and repeatable, as opposed to surgical and biopsy-based cell-stiffness measurements. Techniques such as MRE and ultrasound produce results in real time, providing robust prognostic tools for the benefit of both clinicians and patients^183^. However, current implementations of these non-invasive techniques lack cellular resolution. Hence, widespread techniques like AFM can be used to determine cell stiffness in vitro, with the potential for providing high transverse spatial resolution at the nanoscale^184^. However, this methodology is limited by the need to collect tissue and tumour biopsies. Moreover, even AFM measurements involve methodological variations in, e.g. the cantilever stiffness, shape, and material. This impairs the reliability on the mechanical model used to extract elastic moduli. For instance, Young’s modulus values can vary in a given tissue sample derived from a single patient at different layers of tissue thickness (from 4.89 ± 5.32 to 7.15 ± 6.27 kPa)^185^. Even the geometry of the AFM tip has a measurable effect. Measurements of Young’s modulus for microvessels from the same section gave significantly different values when using a pyramidal tip (67.66 ± 122.26 kPa) or a 1 µm radius spherical tip (8.36 ± 7.55 kPa). This discrepancy likely reflects a heightened sensitivity to the cantilever tips^185^.

A recent study compared the cellular mechanical properties of MCF-7 human breast-cancer cells using seven widely used technologies and showed a 100- to 1000-fold variability in measurements across different methods^186^. This substantial variability in measurement and analysis results highlights the challenge of meaningfully comparing techniques reported across the literature (Table 1). Subtle changes in factors in culture conditions (such as pH, temperature, osmolality, cell passage number) can be a contributor to variations which prevent direct comparison among datasets. Moreover, inconsistencies in the interpretation of biopsy results (patient variations due to lifestyle, gender, age, diversity, etc) and the sampling size of biopsies (representing only about 1/50,000 of the overall tissue^187^) constitute some of the limitations associated with stiffness measurements that may have slowed down their translation to clinical applications. Therefore, a standardised approach for quantifying cell and tissue mechanical properties and establishing a relevant database would be a substantial progress in the field of mechanobiology.

**Table 1:** Stiffness measurements in Bone, Lung, and Brain at tissue/cellular levels

Sample site	Age (yr)	Testing methods	Tissue/Cellular	Young’s modulus (kPa)	Refs.
Bone tissue and cancer					
Excisional biopsy of metastatic cancer from spine or proximal femur	68 ± 15	Stepwise µcompression with time-lapsed µCT imaging	Tissue	Metastatic cancer (*n* = 41)—(201.5 ± 59.68) × 10^3^; Normal specimen (*n* = 61)—(356.2 ± 89.7) × 10^3^	^240^
Specimens from lytic/blastic metastases from distal femoral trabecular bone	45–88	QCT and extensometer modified	Tissue	Lytic (*n* = 2)—(667 ± 121) × 10^3^; Blastic (*n* = 6)—(915 ± 430) × 10^3^; Control (*n* = 19)—(774 ± 360) × 10^3^	^241^
Ewing sarcoma extracted from tibia, humerus and radius of patients	11–26	AFM	Tissue	Control (*n* = 1)—(15.8 ± 1.1) × 10^6^; Patient cases (*n* = 5)—(11.1-13.8) × 10^6^	^242^
Bone (injection of human breast cancer and prostate cancer intracardially in rats)	7 wk old rat	µCT images analysed using intensity-based thresholding algorithm	Tissue	*n* = 15 Healthy vertebrae—7406 × 10^3^; Osteolytic—4550 × 10^3^	^243^
MC3T3-E1 osteoblasts	N/A	AFM	Cellular	G1 phase cells (*n* = 63)—3.5 ± 0.3; S phase cells (*n* = 59)—5.9 ± 0.5	^244^
Lung tissue and cancer					
Human pulmonary arteries (vessel diameter <100 µm) from lung tissues	11–60	AFM	Tissue	Tissue thickness: 10 µm (*n* = 44)—7.15 ± 6.27; 20 µm (*n* = 45)—5.17 ± 4.46; 50 µm (*n* = 40)—4.89 ± 5.32	^185^
Lung cancer patients	N/A	CT images analysed using linear isotropic elastic and neo-Hookean model	Tissue	*n* = 8 Isotropic elastic—1.807; Neo-Hookean—1.346	^245^
Lung tissue from MMTV-PyMT mice	N/A	AFM	Tissue	Lung tissue (*n* = 1)—9.92 ± 4.76; Metastasised lung tissue (*n* = 1)—0.78 ± 0.88	^246^
NSCLC cell lines H23 (adenocarcinoma) and A549 (squamous cell carcinoma)	N/A	Micropipette aspiration	Cellular	H23 cells—0.46 ± 0.18; A549 cells—1.39 ± 0.68	^247^
Pleural effusions from metastatic adenocarcinoma and benign mesothelial cells	N/A	AFM	Cellular	Metastatic tumour (*n* = 40)—0.53 ± 0.10; benign mesothelial cells (*n* = 48)—1.97 ± 0.70	^248^
Brain tissue and cancer					
Mice brain sections were cut centred on the region of lysolecithin injection (corpus callosum)	12 wk old rat	AFM	Tissue	Uninjured contralateral corpus callosum (*n* = 1400)—12.01 ± 6.16; demyelination (post 7 days of injection) (*n* = 1299)—4.34 ± 2.55	^249^
Freshly frozen brain biopsies representing non-tumour gliosis, primary lower-grade gliomas (LGG) and primary glioblastoma (GBM)	N/A	AFM	Tissue	Gliotic tissue ECM (*n* = 5)—0.01–0.18; LGG (*n* = 6)—0.05–1.4; GBM (*n* = 8)—0.07–13.5	^250^
Normal brain parenchyma and brain tumours obtained from patients scheduled for brain tumour removal surgery	24–85	SWE	Tissue	Normal brain tissue (*n* = 63)—7.3 ± 2.1; meningiomas (*n* = 16)—33.1 ± 5.9; low-grade gliomas (*n* = 14)—23.7 ± 4.9; high-grade gliomas (*n* = 18)—11.4 ± 3.6; metastasis (*n* = 15)—16.7 ± 2.5	^251^
Diseased human brain tumours (gliomas, meningiomas lymphomas) and mouse tumours grown in mice and normal mouse brain tissue	N/A	Cantilever-based indenter and analysis using adjusted hertz model	Tissue	Steady-state modulus of human meningiomas (*n* = 118)—3.97 ± 3.66; mouse brain tissue (*n* = 50)—1.56 ± 0.75; human gliomas (*n* = 8)—2.75 ± 1.40; mouse tumour (*n* = 31)—7.64 ± 4.73	^252^
Normal astrocytes and LN229 glioma cells	N/A	AFM	Cellular	Varies with substrate stiffness (*n* = 100–143), for 1 kPa substrate Astrocyte approx. 1.8 ± 0.25; LN229 approx. 1 ± 0.5;	^253^

Some reported mechanical properties of bone, lung and brain tissue and cancer from the literature highlighting the variations in data collection.

## Potential clinical interventions

A metastatic cell engages in heterotypic interactions with different microenvironments that are unique to one organ as it migrates to a secondary organ from the primary tumour site. Therapeutic approaches targeting specific stages of the metastatic cascade therefore hold great promise for cancer patients as they increase the likelihood of combating metastatic disease^188^. Given that TME stiffening contributes to tumour growth and metastasis, one possible approach for therapeutic intervention would be to prevent or reverse this. Targeting matrix crosslinking, a key contributor to tissue stiffness, by inhibiting LOX or related LOXL family members in preclinical ovarian cancer models, has been shown to reduce tumour growth^189^. A recent study on triple-negative breast cancer revealed that hypoxia-mediated LOX targeting can reduce collagen crosslinking and fibronectin assembly, increase drug penetration, and re-sensitise chemotherapy resistance^190^. However, despite promising preclinical models, clinical trials using LOXL2 inhibitors in pancreatic cancer and fibrosis have so far given disappointing results^191,192^. This may be due to insufficient dosing levels for reducing the ECM stiffness, non-specific target tissue engagement in patients, or overcompensation by other pathways mediating collagen crosslinking, including other LOX enzymes. This hurdle can be overcome by employing combinations of therapies that are generally more efficacious. For instance, improvement in the efficacy of anti-angiogenic therapy is achieved by reducing the stiffness of liver metastasis in metastatic colorectal cancer patients^193^.

High internal solid and fluid pressures within a TME prevent drug delivery to the tumour cells. Consequently, enzymes that degrade collagen and hyaluronan (the constituents of the ECM) have been shown to increase the diffusion coefficient of dextran in osteosarcoma xenografts^194^. Hyaluronic acid (HA) has a high capacity for absorbing water and has been shown to impede the intratumoral vasculature in pancreatic ductal adenocarcinoma (PDAC), affecting drug delivery and response in patients^195^. Targeting HA using enzymatic degradation in metastatic PDAC in phase-II clinical trials has improved response rates^196^. However, the most recent reports on clinical phase-III trials (NCT02715804) did not show improvements in the overall survival of metastatic PDAC patients^197^. Hence, the continued development of strategies targeting the ECM can therefore provide powerful tools to monitor tumour progression. Another study targeted pro-fibrotic signals, such as TGF-β1 in breast and pancreatic cancer models, via the angiotensin inhibitor losartan. This resulted in reduced solid stress in tumours by increasing vascular perfusion and decompressing tumour blood vessels while observing reduction in stromal collagen and HA production^198^. An ongoing phase-II clinical trial (ClinicalTrials.gov identifier: NCT03563248) in advanced PDAC patients supports this rationale^199^. Besides, elevated IFP can be targeted by mild systemic heating (39.5 ± 0.5 °C for 4 h) prior to radiation therapy or pulsed focused ultrasound, as observed in human head and neck tumour xenografts which showed increased efficacy in mice and can have potential clinical applications^200^.

Another strategy for mitigating the adverse effects of the stiffened ECM is to suppress the cell response to increased matrix dynamics. The ECM activates integrin-mediated signalling pathways, inducing cellular responses that cause aberrant mechanotransduction at the cell-ECM level via downstream signalling targets^201^. Integrins are known mechanoreceptors and as many as 30 clinical trials are registered that test the effect of integrin inhibitors on cancer progression^201^. The β integrin subunits recruit FAK to initiate downstream signalling cascades, including the Rho, Src, and ERK pathways, and therefore could be potential targets for the cellular response due to the matrix stiffness^202^. Stiffness-activated FAK signalling promotes fibroblast survival by directly inhibiting the apoptosis pathway ^203^. An ongoing clinical trial (NCT02758587) on patients with advanced solid malignancies is investigating the anti-fibrotic effects of the FAK inhibitor^197^. In the meantime, ROCK, a major downstream effector of Rho known to drive cell contractility is a potential target. Significant efforts to develop ROCK inhibitors (fasudil) have not progressed to clinical trials despite successful in vitro results, owing to the pharmacokinetic unsuitability for use in chemotherapy ^204^. Only one ROCK inhibitor (AT13148) has so far progressed to clinical trials (NCT01585701) on patients with advanced cancer treatment. Notably, targeted therapeutics should always be fully characterised in terms of their effects on cancer-cell growth, proliferation, invasion and metastasis, as these factors may not always be positively correlated. For example, the inhibition of cancer proliferation can still promote—rather than block—metastasis^205^.

Physical activity has been identified as an additional therapeutic strategy for reducing cancer patients’ risk of recurrence and mortality. A moderately intense exercise tends to avert tumour spread across the body by normalising angiogenesis, destroying CTCs, and reducing endothelial cell permeability ^206^. Some preclinical reports have indicated that sustained exercise is associated with improved drug delivery and chemotherapy response^207^. Also, an increase in vascular shear stress due to aerobic activity affects the CTC count, which is related to systemic recurrence and mortality in a range of solid tumour types^208^. Shear stresses as high as 60 dynes/cm^2^ (achievable during intensive exercise) can damage or even kill CTCs more effectively than a physiological shear stress of 15 dynes/cm^2,209^. A small pilot study conducted on resected stage I-III colon cancer patients showed reduced CTCs due to exercise^210^. This suggests that exercise can be an important adjunct therapy in cancer management^211^.

Tissue remodelling is common in cancer, and tumour-driven ECM remodelling causes the discharge of ECM components into the bloodstream, the magnitude of which can be a diagnostic or prognostic marker for tumours. Studies have found that collagen IV and MMPs can be used as potential circulating cancer biomarkers in many solid tumours, such as breast, ovarian, and pancreatic cancers^212,213^. Some stroma-derived molecules were uniquely identified as certain oncotypes, for example MMP7 for urological cancer^214^. Hence, the quest for a novel tumour circulatory biomarker (apart from the cancer cell itself) that are easily detectable through high-throughput assays, is a promising new non-invasive diagnostic and prognostic approach.

Amongst the several mutually compensating pro-invasive signalling pathways, cytoskeletal components are identified as non-redundant and attractive targets for blocking cancer invasion^215^. Blocking such cytoskeletal components, such as the myosin IIA family of cytoskeletal motors in glioblastoma, inhibits tumour invasion but enhances tumour proliferation^215^. Recently, mitotic spindles, a crucial player in mitosis, have been shown to evolve during cancer progression, giving a mechanical advantage to metastasis. In other words, several modulators of the spindle length, including TPX2 and kinesin-13, determine the metastatic potential by generating physical forces during cell division to separate the daughter cells from their primary site^216^. Recent studies have provided evidence for the lengthening of mitotic spindles due to upregulated motor protein such as kinesin-5 during cancer progression. This suggests that the spindle aspect ratio could be a clinically important biomarker for highly invasive cancer cells^217^. The newly developing approaches in cancer research have potency to offer fresh avenues for deploying ingenious solutions to explore the development of tractable biomarkers, cell-based stiffness sensors, and innovative cancer therapies that require collaboration between biologists, materials scientists, physicists, and engineers.

## Summary

One of the main advantages of identifying mechanical modulators in the metastatic cascade is the existence of common modalities for most solid tumours. The mechanical signatures can be bifurcated into intra- and extratumoural zones at each step of the metastatic cascade. The intratumoural zone comprises the changes that occur within the cell, while the extratumoural zone encompasses all that is present in the tumour microenvironment, except from the tumour itself. It should be noted that, while this review focuses mainly on the mechanical aspects of cancer progression, these interactions are intrinsically intertwined with the genetic and biochemical alterations that drive cancer outgrowth.

Within the intratumoural zone lies the mechanotransduction response of cancer cells to the changing environmental cues. Because of heterogeneity of cells of the same type in a given region, the cellular response to stress can vary. The mechanical signature of primary tumour growth could be solid stress: compressive in the inner core and tensile at the tumour periphery. The peripheral cells are considered to undergo an unjamming transition (UJT) that liberates them from the dense solid-like phase to a fluid-like unjammed state. Within the cell, several mechanosensors such as integrins and cadherins facilitate force transmission during collective migration, as is commonly observed during cancer invasion. Invading cancer cells also upregulate mechanotransduction regulators such as FAK, ERK, and Rho, and activate mechanosensitive transcription factors YAP/TAZ and MRTF. An increase in tissue stiffness has also been shown to promote persistent glycolysis and an imbalance of cellular circadian rhythm in the tumour epithelia, when compared to normal epithelia^218,219^. Altered cellular metabolism is observed in migrating cancer cells through a collagen matrix. For instance, the ATP:ADP ratio decreases in cancer cells in an aligned matrix while the cells utilise more ATP (ATP:ADP higher) to migrate through denser matrices. Besides, cell invasion promotes cytoskeleton remodelling, with some cells undergoing EMT leading the remaining proliferative non-EMT cells to intravasation. Such EMT cells show increase in intracellular viscosity, stress fibres, focal adhesions, F-actin assembly turnover, contractility, and exerting high traction forces to navigate through the ECM. To allow intravasation, cancer cells need to display enhanced cellular and nuclear deformability and membrane fluidity to cross endothelial cell-cell junctions. At this stage, the cells have a disorganised cytoskeleton, reduced stiffness, increased plasticity, and anchorage-independent motility. Naturally, cells with a small nuclear volume have an added advantage for intravasation. As CTCs, circulating as clusters constitutes survival advantages through quick and easy intravascular arrest in capillaries. These cells have increased surface glycoproteins with which to form firm adhesions to endothelial walls. They also show reduced actomyosin contractility, reduced F-actin assembly, and cellular softening, besides withstanding high fluid shear stress. Shear stress also activates EMT and YAP/TAZ transcription factors during extravasation. In addition, cancer cells either form invadopodia or influence endothelial cells to form dome-shaped endothelial arcs to initiate exit from blood vessels, thus making way for non-EMT cells or for EMT/MET transformed cells. After exiting, cancer cells actively compete with the pericyte lining of the vascular wall, and epithelial cancer cells restart growth at the secondary site. The migrated cancer cells adapt to the new substrate stiffness based on their mechanical memory mediated by YAP and differential expression of miR-21 that regulates cellular force generation.

The extratumoural zone comprises solid and fluid components in the tumour niche, including interstitial spaces, the ECM, and neighbouring cells. During primary tumour growth, the mechanical signature can be summarised as enhanced IFP, outward interstitial fluid velocity, vessel compression, tissue stiffening, increased vascular permeability, and enhanced pro-tumour activity of tumour associated cells. There are alterations in ECM components such as hyaluronic acid contributes to IFP, and excessive collagen, laminin, fibronectin deposition majorly affects tissue stiffness. The collective involvement of these factors determines the metastatic potential of cancer. For instance, a decrease in ECM confinement triggers cancer invasion. Besides, the physical interaction of stromal cells (e.g. fibroblasts, adipose-derived stromal cells) in cancer invasion has been identified. More research to ascertain the physical involvement of other cell types would provide new insights for novel interventions. This is because force transmission through direct cell contacts is more powerful and quicker than biochemical gradients for influencing migration. The signature associated with intravasation could be endothelial junction openings, causing vascular leakiness. During systemic circulation, the blood-flow profile (regions with low wall shear stress) and narrow vessel geometries are mechanical cues that govern cell arrest and secondary sites. The shear-stress pattern also influences the receptor-ligand binding of CTCs to the vessel wall for further extravasation. Some cases of CTC clusters have been observed to bring components of the primary tumour region (fibroblasts, soluble factors) to increase familiarity in an unfamiliar secondary site, accelerating metastatic onset. Thus, measures to break up a CTC cluster could be an adjunct intervention strategy. Overall, it can be said that the uncontrolled proliferation of cancer cells challenges the force balance between neighbouring microenvironments, which initially resist as well as support tumour growth in a complex manner.

## Discussion and future perspectives

The transition of cancer cells from a localised region of growth (often curable) to their dissemination to a distant organ (mostly incurable) underlies most cancer-related deaths. The recurrence of cancer and late-stage diagnosis are currently treatable but not curable. Hence, a comprehensive understanding of the mechanisms underpinning cancer metastasis is essential. In this review, we discussed the complex interrelationship between biochemical and mechanical cues in the cellular microenvironment. We ascertained that cells actively probe the mechanical properties of their environment and respond dynamically by altering their behaviour accordingly through mechanotransduction signalling. We also noted the need for standardising the documentation of mechanical measurements at the cellular and tissue levels^220^. While general ranges of Young’s modulus can be obtained for healthy and cancerous tissue in various organs, the challenge of achieving the require precision precludes the determination of a quantifiable relationship between the metastatic potential and tissue/cell stiffness. The disparities in experimental parameters and variables in published studies complicates data analysis considerably. Factors such as the patient age, medical history, lifestyle, cell and tissue heterogeneities, and sample properties (including size, shape, preservation methods, testing timelines, analysis methods, and tissue abnormalities) are major sources of variability between measurements; yet they are often inconsistently reported. The repeatability and comparability of mechanical measurements under a given set of conditions is therefore crucial for translation to medical assessment. Some inconsistencies related to instrumentation, sample processing methodologies, and analysis can be resolved by standardisation. Databases can be broadened by including demographic details. New research must report detailed experimental methodologies and the associated force profiles in mechanical measurements. In addition, reports should specify the underlying assumptions used in analytical models, such as the type of behaviour (e.g. linear elasticity for AFM or viscoelasticity) or the value of Poisson’s ratio. This is important to limit errors when inferring material properties from primary data. Similarly, reporting common cell properties (e.g. their morphology), evaluating biomechanical properties (especially the elasticity, deformability, and TME properties—IF, ECM properties, etc.) in cancer research studies would serve to develop a reliable and reproducible database to effectively correlate to disease phenotypes. A standardised operational protocol (SOP) for AFM was validated across six EU laboratories to quantify biomechanical properties independently of the instrument used, the cell culture conditions, and the data analysis^221^. Using more of such SOPs will enable morpho-mechanical properties to serve as label-free biophysical markers, identifying functional processes^222^.

Throughout this review, we discussed the process of cancer metastasis in terms of the mechanical alterations and the physical barriers that trigger the metastatic journey of cancer. We described the continual biophysical adaptation of cancer cells. An increasingly important emerging concept is that of mechanical memory, whereby cells retain a memory of their interaction with an earlier mechanical cue. However, the extent of this memory is influenced by the magnitude and duration of a mechanical stress capable of reprogramming the cell via persistent epigenetic changes^223^. More research is needed to determine whether the mechanical memory gained during biophysical adaptions in a primary TME primes cells to endure a secondary TME^224^. Designing therapeutic inhibitors of epigenetic modifiers to disrupt mechanical memory can potentially aid in the discovery of a new class of anti-metastatic drugs.

Recent advances combining mechanical measurements with single cell sequencing methods will aid researchers to understand the causes and consequences of biophysical signatures in cancer and their relationship with the biological hallmarks of cancer. New single-cell imaging methods provide insights into the cellular context of ECM remodelling and cell-ECM interactions at the single-cell and subcellular levels. High-resolution tissue imaging probes the spatial and temporal cell-ECM interactions. Multiplexed ion-beam imaging (MIBI), a novel technology, can quantitatively map the proteomic landscape of single cells in primary tumours and metastases^220^. Such multiplex protein imaging and analysis were applied successfully to clinical cohorts, as reviewed by Souza et al.^225^. A combination of AFM-based single-cell mechanics measurements and RT-qPCR-based gene-expression analysis on the same cell revealed a correlation between pro-metastatic gene expression and soft single cells^226^. Another new strategy involves an electroporation-based lipid-bilayer assay called ELASTomics that can profile single-cell mechanical and molecular phenotypes^227^. It presents the benefit of jointly examining the cell surface mechanics and the underlying transcriptional regulation with an unprecedented degree of resolution owing to its easy integration with existing single-cell sequencing methods. This highlights the increased interest, within the scientific community, to innovate and develop more mechanical measurement methods. More such models or tools are needed, for instance, to differentiate between the biological consequences of solid stress and other factors, such as increased stiffness. Early evidence in HCC patient cohorts identified stiffness as an independent predictor of late tumour recurrence^228^. Thus, detailed research is needed to clarify the role played by the physical characteristics of cancer and its interaction with the secondary TME with regard to the recurrence of tumour and the development of resistance to therapies.

As a cell adapts to changes in its microenvironment, it undergoes a dynamic metabolic regulation that is less heterogeneous than the genetic landscape of tumours. This may underlie an appealing anticancer therapy strategy ^229^. A link between the cell migration mode and its energy metabolism has been noted^230^. Recent studies have revealed cancer cells’ adaptive choices for conserving energy and migrating securely in response to oxygen and energy deprivation^69,231^. However, more studies are required to identify the metastasis-associated energy-conserving mechanisms to pinpoint the metabolic vulnerabilities for molecular intervention. Cancer dissemination might thus be countered by targeting mechanisms shared by metabolic adaptation, migration signalling, anti-apoptosis processes, stemness, and survival^232^.

The recent influx of therapeutic interventions mitigating the effect of increased ECM stiffness entering clinical trials highlights the therapeutic potential of mechanical regulation. Parallels can also be drawn between solid and liquid tumours with respect to mechanical signatures. Physical constriction through cell overcrowding, either in the vasculature or in the bone marrow (resulting in limited oxygen and nutrients in the microenvironment), requires drastic mechanical adaptations by cancer cells; this phenomenon remains to be explored^233^. The manipulation of biophysical characteristics, the identification of novel blood biomarkers, and the therapeutic value of moderately intense physical activity have emerged as avenues for future development^214^. Previously defined biophysical markers would continue to be identified and refined with an increased awareness, in parallel with the development of techniques, considering the essential mechanical aspects of the process involved^233–235^. As highlighted, despite the surge in progress in this area over the past two decades, this success is far from satisfactory, as witnessed by the seriousness of cancer metastasis and the devastating impact on patients’ longevity and quality of life. Investment and further research into the role of mechanics in metastatic progression may provide new avenues for developing breakthrough treatments benefitting late-stage cancer patients.

## Data Availability

No datasets were generated or analysed during the current study.
